# Amyloid-β 1–24 C-terminal truncated fragment promotes amyloid-β 1–42 aggregate formation in the healthy brain

**DOI:** 10.1186/s40478-016-0381-9

**Published:** 2016-10-10

**Authors:** Sonia Mazzitelli, Fabia Filipello, Marco Rasile, Eliana Lauranzano, Chiara Starvaggi-Cucuzza, Matteo Tamborini, Davide Pozzi, Isabella Barajon, Toni Giorgino, Antonino Natalello, Michela Matteoli

**Affiliations:** 1 IRCCS Humanitas, via Manzoni 56, 20089 Rozzano, Italy; 2 Hertie Institute and Deutsches Zentrum für Neurodegenerative Erkrankungen (DZNE), Otfried-Müller-Straße 27, 72076 Tübingen, Germany; 3 Humanitas University, via Manzoni 56, 20089 Rozzano, Italy; 4 IN-CNR, Corso Stati Uniti 4, 35126 Padova, Italy; 5 Department of Biotechnology and Biosciences, University of Milano-Bicocca, Piazza della Scienza 2, 20126 Milano, Italy; 6 IN-CNR, via Vanvitelli 32, 20129 Milano, Italy

**Keywords:** Amyloid-β, Alzheimer’s disease, Microglia, Proteolytic activity, Aβ24

## Abstract

Substantial data indicate that amyloid-β (Aβ), the major component of senile plaques, plays a central role in Alzheimer’s Disease and indeed the assembly of naturally occurring amyloid peptides into cytotoxic aggregates is linked to the disease pathogenesis. Although Aβ42 is a highly aggregating form of Aβ, the co-occurrence of shorter Aβ peptides might affect the aggregation potential of the Aβ pool. In this study we aimed to assess whether the structural behavior of human Aβ42 peptide inside the brain is influenced by the concomitant presence of N-terminal fragments produced by the proteolytic activity of glial cells. We show that the occurrence of the human C-terminal truncated 1–24 Aβ fragment impairs Aβ42 clearance through blood brain barrier and promotes the formation of Aβ42 aggregates even in the healthy brain. By showing that Aβ1-24 has seeding properties for aggregate formation in intracranially injected wild type mice, our study provide the proof-of-concept that peptides produced upon Aβ42 cleavage by activated glial cells may cause phenotypic defects even in the absence of genetic mutations associated with Alzheimer’s Disease, possibly contributing to the development of the sporadic form of the pathology.

## Introduction

Alzheimer’s disease (AD) is a protein misfolding pathology, caused by accumulation of abnormally folded Aβ and tau polypeptides, which form amyloid plaques and neurofibrillary tangles in the brain of affected individuals. Aβ aggregates have been linked with learning and memory deficits in both human and mouse models of the disease, making Aβ deposition a target for prevention and treatment [1–3]. In the last years, a lot of effort has been focused on the identification of the processes leading to Aβ aggregation. Evidence have indicated that, although Aβ42 is a highly aggregating form of Aβ [4, 5], the co-occurrence of Aβ peptides with different length can affect the neurotoxic and aggregation potential of the Aβ pool (reviewed in [6]). As an example, changes in the ratio of Aβ40/42 has been shown to represent an important factor in initializing Aβ fibrillogenesis and toxicity [7], indicating that the presence of different Aβ forms may affect the development of AD in vivo.

Consistently, while small amounts of Aβ-containing brain extracts, deriving from either AD patient or AD transgenic mouse, induce β-amyloidosis and glial activation once intracranially injected in pre-depositing AD transgenic mice [8–11], the chronic infusion of soluble, synthetic Aβ42 peptides into wild type (wt) rodent brains does not result in amyloid deposition [9]. The finding that Aβ42 alone fails to show seeding properties in the healthy brain and does not trigger pathogenetic pathways indicates the occurrence of efficient clearance mechanisms and suggests that brain-specific cofactors, specifically present in pathological conditions, are needed for effective seeding [9].

Although aggregation properties of full length Aβ42 have been deeply explored both in vitro and in vivo, much less is known about the in vivo aggregating properties of shorter Aβ fragments. This aspect may be particularly relevant, considering that N-terminal fragments of different length are largely produced by the proteolytic activity of glial cells during the development of AD [12]. Indeed, several proteases including neprilysin [13], insulin-degrading enzyme [14], endothelin-converting enzyme [15], angiotensin-converting enzyme [16] and matrix metalloproteinase-9 (MMP9) [17–20] have been shown to degrade soluble Aβ in vitro, acting at specific cleavage sites and generating characteristic Aβ fragments. These proteolytic activities, therefore, are critical in determining the quantitative and qualitative pattern of cerebral Aβ levels ([21]; reviewed in [22]). MMP9 in particular, which generates different C-terminal truncated Aβ fragments, including 1–16, 1–20, 1–23, 1–30, 1–33 and 1–34 [19, 23], is thought to play relevant roles in different pathological contexts, as suggested by the observation that its expression can be stimulated by diverse insults, including Aβ itself [24], and is up-regulated in glia cells adjacent to amyloid deposits [25].

Since Aβ N-terminal fragments of varying lengths are expected to exhibit different physico-chemical properties which may result in different aggregation behaviors, as also indicated by modeling of aggregation determinants with bioinformatics methods [26], we aimed to investigate whether dynamics of interaction and structural behavior of human Aβ42 peptide inside the brain are influenced by the concomitant presence of C-terminal truncated fragments. We took advantage of the commercially available synthetic human Aβ1-24 peptide (referred to as Aβ24), a C-terminal truncated Aβ fragment overlapping with MMP9 cleavage products (residues 1–20 and 1–23, [20]) and coincides with a turn region between β-sheets in recently-resolved fibrillar structures. Our results indicate that the presence of Aβ24 in intracranially injected wild type mice impairs Aβ42 clearance and promotes formation of Aβ42 aggregates even in the healthy brain.

## Results

### Synthetic Aβ24 fragments promote aggregates formation in wt mice brain

**Fig. 1:**
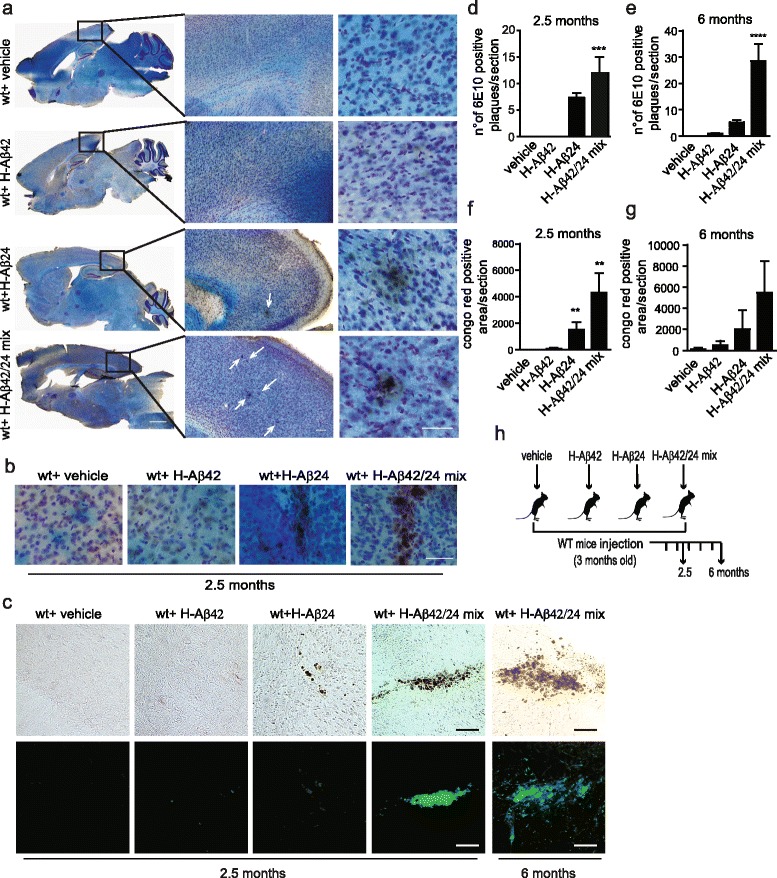
Intracranial injection of H-Aβ24 and H-Aβ42/H-Aβ24 mix in wt mice induces amyloid aggregate deposition. **a** 6E10 DAB staining of hippocampal brain sections 6 months after vehicle, H-Aβ42, H-Aβ24 or H-Aβ42/H-Aβ24 mix injection. Sagittal plane sections are shown on the left; middle and right panels show enlargements of the dorsal cerebral cortex. 6E10-immunopositive aggregates are visible on a cresyl *violet*-luxol fast *blue* counterstaining. Scale bars: left 1 mm, middle 100 μm, right 50 μm. **b** 6E10 DAB staining of brain sections 2.5 months after vehicle, H-Aβ42, H-Aβ24 or H-Aβ42/H-Aβ24 mix injection. Scale bar: 50 μm. **c** Congo *red* staining of brain sections 2.5 months after the injection of vehicle, H-Aβ42, H-Aβ24 or H-Aβ42/H-Aβ24 mix. On the right, representative images of aggregates detected 6 months after H-Aβ42/H-Aβ24 mix injection are shown. Top: bright field images; bottom: epifluorescence images -FITC filter. Scale bar: 50 μm. **d** and **e** Quantification of 6E10 DAB positive plaques 2.5 or 6 months after the injection of vehicle, H-Aβ42, H-Aβ24 or H-Aβ42/H-Aβ24 mix. 6 brain sections were analyzed for each mice (*N* = 3 mice for each group). Statistical analysis was performed by One way Anova, Bonferroni multiple comparison test (****P < 0.0001; ***P < 0.001). **f** and **g** Quantification of Congo *red*-positive plaques area (μm^2^) per brain section at 2.5 or 6 month after injection. 10 sections of 50 μm thickness per slice were analyzed for each mouse brain (*N* = 6 mice for each group). Statistical analysis was performed by One way Anova, Bonferroni multiple comparison test (**P < 0.01). **h** Cartoon depicting the experimental scheme

Three month-old wt mice were intracranially (i.c.) injected with either the oligomeric form of the single H-Aβ42 peptide or with an equimolar mixture of oligomeric H-Aβ42 and H-Aβ24 peptides, and the brains were examined after 2.5 or 6 months (see cartoon, Fig. 1h). As previously described [9], H-Aβ42 injected in the brain of wt mice and examined 6 months later did not cause Aβ deposition. H-Aβ24, and even more potently H-Aβ42/H-Aβ24 mix, induced the formation of aggregates, detected with the Aβ N-terminal specific antibody 6E10, followed by HRP (Fig. 1a and e). 6E10-positive aggregates were already detectable 2.5 months after injection of H-Aβ24 or H-Aβ42/H-Aβ24 mix in the mice brains (Fig. 1b and d). Aggregates were also detectable by the amyloidogenic dye Congo red both 2.5 and 6 months after injection (Fig. 1c and f, g). Consistently, the highest number of Congo red positive aggregates was detected in the brains of mice injected with H-Aβ42/H-Aβ24 mix (Fig. 1f and g).

**Fig. 2:**
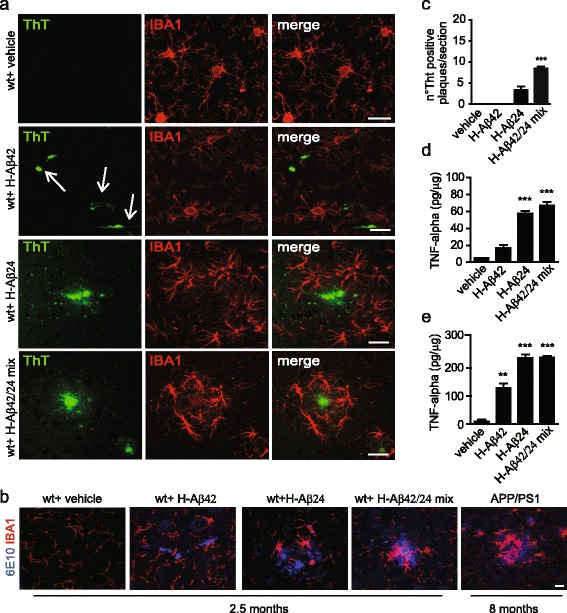
Intracranial injection of H-Aβ24 and H-Aβ42/H-Aβ24 mix in wt mice induces microglia recruitment and TNF-alpha production. **a** Immunofluorescence staining of ThT-positive aggregates (*green*) surrounded by Iba1 positive microglia cells (*red*) in wt mice brain slice 2.5 months after injection of vehicle, H-Aβ42, H-Aβ24 or H-Aβ42/H-Aβ24 mix. Arrows in H-Aβ42 panel indicate Aβ accumulation in vessels. Scale bar: 10 μm. **b** IF staining of 6E10 positive aggregates (*blue*) surrounded by Iba1 positive microglia cells (*red*) in wt mice brains 2.5 months after injection of vehicle, H-Aβ42, H-Aβ24 or H-Aβ42/H-Aβ24 mix. IF staining of 6E10 positive plaques and surrounding microglia are shown in a brain section of 8 months old APP/PS1 transgenic mouse for comparison. Scale bar: 10 μm. **c** Quantification of ThT-positive plaques in sections of wt mice brains 2.5 months after injection of vehicle, H-Aβ42, H-Aβ24 or H-Aβ42/H-Aβ24 mix. Aβ detected in correspondence of blood vessels (H-Aβ42 *left panel*, *arrows*) was excluded from the analysis. 6 sections were analyzed for each mouse brain (*N* = 3 mice for each group). Statistical analysis was performed by One way Anova, Bonferroni multiple comparison test (*** *P* < 0.001). **d** and **e** TNF-alpha levels measured by ELISA in brain homogenates **d** and serum **e** of wt mice 2.5 months after the injection of vehicle, H-Aβ42, H-Aβ24 or H-Aβ42/H-Aβ24 mix. Statistical analysis was performed by One way Anova, Bonferroni multiple comparison test (*** *P* < 0.001; ** *P* < 0. 01)

**Fig. 3:**
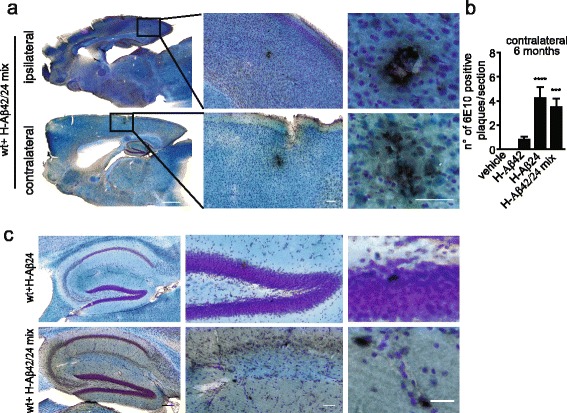
Intracranial injection of H-Aβ42/H-Aβ24 mix in wt mice induces spreading of misfolded Aβ. **a** 6E10 DAB staining of hippocampal brain sections 6 months after H-Aβ42/H-Aβ24 mix injection. Sagittal plane sections are shown on the left; middle and right panels show enlargements of cerebral cortex; the injected hemisphere is referred as ipsilateral (*top panel*) and the non-injected one as contralateral (*bottom panel*). 6E10-immunopositive aggregates are visible on a cresyl *violet*-luxol fast *blue* counterstaining. Scale bars: left 1 mm, middle 100 μm, right 50 μm. **b** Quantification of 6E10 DAB positive plaques in the contralateral hemisphere 6 months after the injection of vehicle, H-Aβ42, H-Aβ24 or H-Aβ42/H-Aβ24 mix. 6 brain sections were analyzed for each mouse (*N* = 3 mice for each group). Statistical analysis was performed by One way Anova, Bonferroni multiple comparison test (**** *P* < 0.0001, *** *P* < 0.001). **c** Representative images of 6E10 DAB positive aggregates at the hippocampal level 6 months after Aβ injection. Scale bar: left and middle 100 μm, right 50 μm

As a further confirmation for the formation of amyloidogenic aggregates, brain sections from mice i.c. injected and incubated for 2.5 months were stained with thioflavin T (ThT), a benzothiazole dye that exhibits enhanced fluorescence upon binding to amyloid fibrils [27, 28]. ThT-positive aggregates were detected in wt mice brain injected with H-Aβ24 or with H-Aβ42/H-Aβ24 mix (Fig. 2a and c); both ThT- (Fig. 2a) and 6E10- (Fig. 2b) positive aggregates were surrounded by microglia, as revealed by Iba1 staining (Fig. 2a and b, red staining). Again, no ThT positive aggregates were detected in vehicle-injected wt mice, whereas only ThT-positive blood vessels, which are not surrounded by microglia, were visible in wt mice injected with H-Aβ42 (Fig. 2a, arrows). The recruitment of microglia around aggregates was reminiscent of the plaques present in APP/PS1 transgenic mice brain (Fig. 2b, right). Consistent with data reported in AD patients and mice models [29–33], a significant increase in the levels of tumor necrosis factor-alpha (TNF-alpha) was detected by ELISA in the serum and brains of mice i.c. injected with H-Aβ24 and of H-Aβ42/H-Aβ24 mix, and much less with H-Aβ42 alone (Fig. 2d and e). These data indicate that H-Aβ24 and, even more prominently, H-Aβ42/H-Aβ24 mix induce an inflammatory reaction in the brain of injected mice. Interestingly, 6 months after i.c. injection, few scattered 6E10-positive spots also start to become detectable in the non-injected side (Fig. 3a and b), suggesting a possible spreading of misfolded/aggregating Aβ. Also, amyloid aggregates were detectable at the hippocampal level in the injected hemisphere (Fig. 3c).

### Behavioral deficits in wt mice injected with Aβ24 peptide

**Fig. 4:**
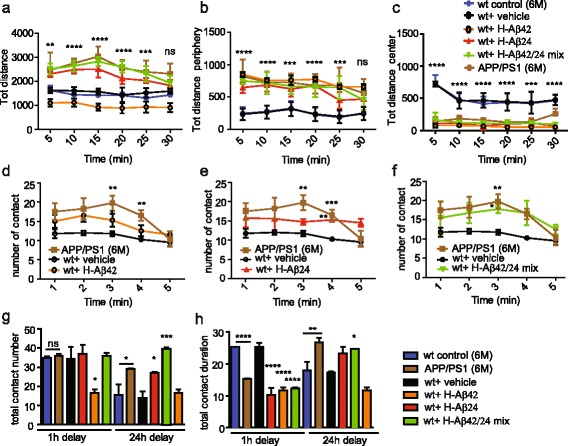
Intracranial injection of H-Aβ induces behavioral defects in wt mice. **a**–**c** Mice hyperlocomotion, assessed by Open Field test. Left panel **a** total distance (cm) travelled by mice in 30 min using 5 min time bin representation. **b**, **c** panels: total distance travelled in 30 min in the periphery or in the center of the arena using 5 min time bin representation. Statistical analysis was performed by One way Anova, Bonferroni multiple comparison test. Asterisks are referred to the vehicle vs H-Aβ42/H-Aβ24 mix condition. No significant differences are present between APP/PS1 tested mice and the H-Aβ42/H-Aβ24 mix condition. (****P < 0.0001; ***P < 0.001; **P < 0.01). **d**–**f** Social interaction, assessed using the social free test. Graphic representation of the number of contacts/min between the two mice in the same arena. H-Aβ42 **d**, H-Aβ24 **e** or H-Aβ42/H-Aβ24 mix **f** are compared to vehicle injected mice and APP/PS1 mice. Statistical analysis was performed by One way Anova, Bonferroni multiple comparison test (**P < 0.01; *P < 0.05). **g** and **h** Assessment of learning and memory by NOR. **g** Number of contacts with the new and old object either after 1 h and 24 h of delay. **h** Number of contacts with the new object both after 1 h and 24 h of delay. Statistical analysis was performed by One way Anova, Bonferroni multiple comparison test. Asterisks are referred to the control wt vs APP/PS1 mice when specified or to the vehicle vs other conditions (****P < 0.0001; **P < 0.01; *P < 0.05). A total of 33 mice (6 mice for each experimental group plus 6 APP/PS1 and 3 wt mice 6 months old) were analysed

To investigate whether the formation of Aβ aggregates and the increase in TNF-alpha were accompanied by the occurrence of cognitive defects [34–36], mice were analysed using 3 different behavioral tests: the open field, a recognized paradigm for assessing motor activity and anxiety-like behaviors in response to a novel environment [37–39]; the sociality task, which also unveils anxious and aggressive behaviors; and the novel object recognition (NOR), that, monitoring the time spent by mice to explore a novel object, enables the assessment of possible declines in learning and memory. Consistently with literature data [40], 6 months old APP/PS1 mice displayed significant hyperactivity and anxiety compared to the wt littermates, as shown by both the open field and sociality tasks (Fig. 4a–f). Interestingly, wt mice injected with the mix of the two peptides displayed abnormal behavioral phenotypes very similarly to age matched APP/PS1 mice, both in terms of enhanced motor activity, as indicated by the total distance traveled (Fig. 4a), and in terms of increased anxiety levels, as revealed by the longer time spent in the periphery (Fig. 4b) and the shorter time spent in the center (Fig. 4c) of the arena. This result was also confirmed by the sociality task in which APP/PS1 and wt Aβ-injected mice had a higher number of contact and spent less time with the second animal (Fig. 4d–f). In the NOR test, APP/PS1 mice as well as wt mice injected with the mix of the two peptides spent less time than wt littermates exploring the novel object at the 1 h delay test and more time exploring the novel object at the 24 h delay test, indicating a significant defect in learning and memory (Fig. 4g and h). Although not displaying brain aggregates, some behavioral alterations were detected in mice intracranially injected with oligomeric H-Aβ42, consistent with literature data [41, 42]. Conversely, no differences were noticed between the not injected and vehicle injected wt mice, showing that the injection per se was not responsible for the observed behavior defects. These data indicate that injection of H-Aβ24 and, even more prominently, H-Aβ42/H-Aβ24 mix into wt brain results in the occurrence of cognitive defects comparable to APP/PS1 mice.

### Predicted physico-chemical properties of Aβ24 aggregation and cross-aggregation

We next aimed at assessing the molecular basis of aggregate formation in the brains injected with H-Aβ24 or with the H-Aβ42/H-Aβ24 mix. In order to understand whether H-Aβ24 is endowed with different propensity to form aggregates relative to H-Aβ42, we modeled the determinants of aggregation with bioinformatics methods, using different algorithms which predict the aggregation propensity on the basis of sequences [26]. First, the AMYLPRED2 consensus analysis identified two distinct amyloidogenic regions in the full Aβ42 peptide, respectively encompassing residues 15–22 and 29–42. Of the two regions, the first was predicted in Aβ24 as well (Table 1). This is an indication that Aβ24 maintains fibrillogenic potential in isolation [43], although the resulting fibrillar structure will likely be different due to the lack of second aggregation-prone region.

**Table 1:** Amyloigenic regions in Aβ24 and Aβ42 peptides predicted by the 11 indicated methods, and AMYLPRED2-derived consensus score

Method	Aβ24	Aβ42
AGGRESCAN	17–22	17–22, 30–42
AmyloidMutants	4–12, 15–23	14–22, 34–42
Amyloidogenic Pattern	16–21	16–21
Average Packing Density	16–21	16–21, 32–37
Beta-strand contiguity	15–20	15–20, 29–41
Hexapeptide Conf. Energy	16–22	16–22, 29–42
NetCSSP	1–23	1–23, 28–37
Pafig	7–24	7–42
SecStr	15–20	15–20
TANGO	17–21	17–21, 29–41
WALTZ	15–24	15–23, 28–42
**AMYLPRED2 (CONSENSUS5)**	**15–22**	**15–22, 29–42**

**Fig. 5:**
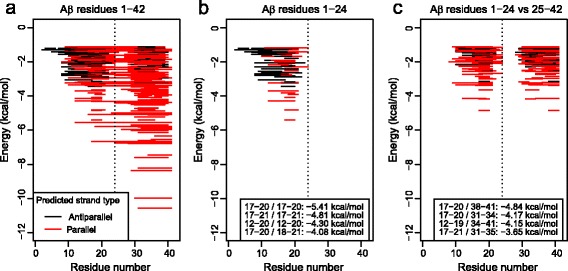
Bioinformatics analysis of Aβ42, Aβ24 and cross-aggregating regions and their stabilities. Regions and energies are predicted by Walsh’s PASTA 2.0 algorithm; lower energies indicate higher fibrillation propensity. **a** Self-aggregation propensities for the full length Aβ42 protein; **b** self-aggregation propensities for the Aβ24 fragment. **c** Cross-aggregation between the Aβ24 fragment and the C-terminal region (aa 25 to 42) of Aβ42

Second, the PASTA 2.0 algorithm by Walsh et al. [44] predicted the strongest self-aggregating segment in Aβ42 to be residues 31–41 (at about−10.6 kcal/mol, (Fig. 5a), thus estimating Aβ24 to be both a weaker self-binder (by approximately 5 kcal/mol) and to have a comparatively more marked, although still not dominant, tendency towards the self-assembly in the antiparallel arrangement (Fig. 5b). It is worth noting that the algorithm also predicts cross-aggregation propensities between segments in the 1–24 and 25–42 ranges, the strongest pair being at−4.84 kcal/mol, which is in the same order of magnitude of the self-aggregation propensities in the 1–24 region (Fig. 5c).

### Aβ24 displays a low fibrillar aggregation propensity and supports the formation of intermolecular β-sheets

**Fig. 6:**
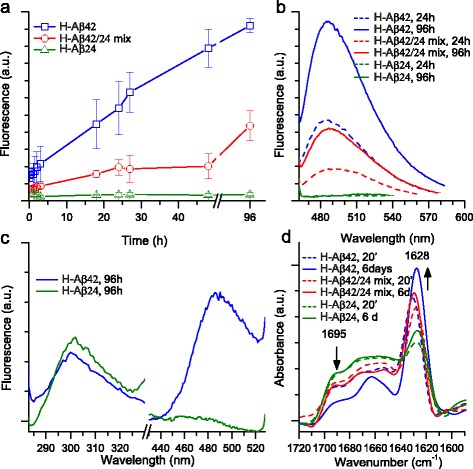
In vitro aggregation of Aβ peptides studied by ThT fluorescence and infrared spectroscopy. **a** The time course of aggregation of Aβ42, of Aβ24, and of the equimolar mixture of the two peptides incubated at 37 °C in PBS was monitored by ThT fluorescence with excitation and emission wavelengths at 450 nm and 485 nm, respectively. **b** ThT fluorescence emission spectra (excitation at 450 nm) of samples as in a) reported for selected incubation times. **c** Fluorescence emission spectra with excitation at 270 nm of Aβ42 and of Aβ24 incubated at 37 °C in PBS for 96 h. **d** ATR-FTIR spectra of Aβ42, of Aβ24, and H-Aβ42/H-Aβ24 mix incubated at 37 °C in PBS for different times, as indicated. Spectra are reported after Fourier self deconvolution (see Materials and Methods)

The in vitro aggregation of Aβ samples was monitored by ThT assay [45] (Fig. 6a–c). ThT fluorescence emission was recorded at different incubation times at 37 °C in physiological buffer. Under our experimental conditions, and in agreement with previously reported data [46], oligomeric H-Aβ42 rapidly formed ThT-positive aggregates already after 24 h, while oligomeric H-Aβ24 displayed very low ThT fluorescence up to 96 h of incubation (Fig. 6a and b), indicating a much lower fibrillation capability than H-Aβ42. Further, we recorded the emission fluorescence spectra of the samples incubated for 96 h at 37 °C after tyrosine excitation at 270 nm, which is also able to produce ThT fluorescence [47]. The tyrosine emission at around 301 nm was detected for both H-Aβ24 and H-Aβ42 samples, but only the latter showed the ThT emission typical of amyloid fibrils at around 485 nm (Fig. 6c). An intermediate behavior was observed for the equimolar mixture of oligomeric H-Aβ42/H-Aβ24 peptides (Fig. 6a and b). The secondary structures of the peptide assemblies were also investigated by Fourier transform infrared (FTIR) spectroscopy in attenuated total reflection (ATR) [48, 49] (Fig. 6d). The FTIR spectra of the H-Aβ24 samples incubated at 37 °C for 20 min and for 6 days were characterized by a broad Amide I band, due to the C = O stretching vibrations of the peptide bond, with two main peaks at ~1695 cm^−1^ and ~1628 cm^−1^, assigned to the formation of intermolecular β-sheet structures. In comparison to H-Aβ24, H-Aβ42 at 20 min displayed a higher intensity of the ~1628 cm^−1^ peak. During incubation at 37 °C of H-Aβ42, this component increased in intensity while that at ~1695 cm^−1^ decreased (Fig. 6d). These spectral changes have been already observed in the fibrillogenesis of Aβ peptides and of other proteins, and assigned to the conformational conversion towards fibrillar structures with a parallel orientation of the intermolecular β-sheets [48, 49]. An intermediate behavior was observed for the equimolar H-Aβ42/H-Aβ24 mix (Fig. 6d). Therefore, the spectroscopic analyses indicated a low fibrillization propensity for H-Aβ24 and confirmed the higher antiparallel content of this peptide, as predicted by bioinformatics analysis (Fig. 5c). These data, together with the cross-aggregation potential between H-Aβ1-24 and H-Aβ25-42 (Fig. 5c), suggest that H-Aβ24 may promote aggregate formation involving intermolecular β-sheet interactions, which possibly retain H-Aβ42 through cross-aggregation between segments in the 1–24 and 25–42 ranges.

### Synthetic Aβ24 fragment impairs Aβ42 clearance in a blood brain barrier model

**Fig. 7:**
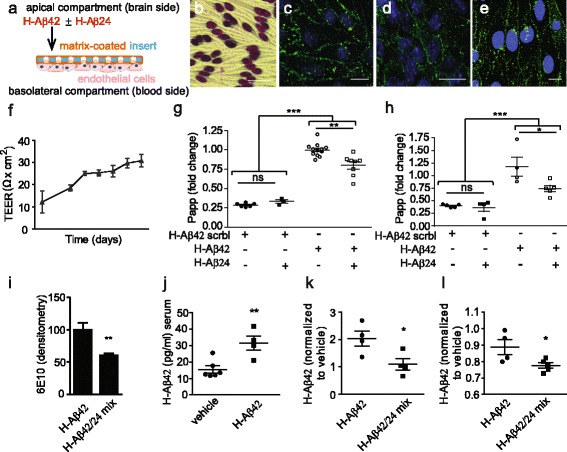
Aβ24 fragment diminishes Aβ42 clearance through the BBB. **a** Graphical representation of the in vitro BBB model composed by a monolayer of brain endothelial cells seeded and cultured on an inverted matrix-coated porous membrane, allowing an apical compartment (donor-“brain”-side) physically separated from the basolateral chamber (receiving-“blood”-side). **b** Representative images of brain endothelial cells tightly wedged together and **c** expressing cell type-specific tight junctional proteins claudin-5 (*green*) **d** and ZO-1 (*green*), and **e** the gap junction protein Connexin-43 (*green*). Nuclei counter-stained with DAPI (*blue*). Scale bars: 20 μm. **f** Barrier properties monitored in terms of gradual increase in transendothelial electrical resistance (TEER) during cell monolayer’s formation over time. **g** and **h** Apical-to-basolateral exchange across endothelial monolayer of fluorescent Aβ42 or scramble Aβ42 control peptide, 1 μM g) or 100 nM h), over 120 min in presence or absence of Aβ24 at equimolar concentration. Quantification of unidirectional trans-endothelial Aβ42 transport by fluorescence spectrophotometry *n* ≥ 3 experiments; statistical analysis was performed by One way Anova, followed by Bonferroni’s post hoc test for multiple comparisons (**P < 0.01; ***P < 0.001). **i**) Dot blot analysis of medium collected in the abluminal compartment 120 min after brain endothelial cell monolayers exposure to Aβ42 or Aβ42/Aβ24 mix. Histograms represent the densitometry quantification upon staining with anti-6E10 antibody. Results are expressed as mean values of triplicates in each experimental group ± SE. Values were normalized on control; statistical analysis was performed by unpaired T test (**P < 0.01). **j** Aβ42 (pg/ml) absolute values detected by ELISA in the serum of mice 1 week after H-Aβ42 intracranical injection. **k** Aβ42 serum level measured 1 week after the injection of vehicle, H-Aβ42 and H-Aβ42/H-Aβ24 mix (2pmol of H-Aβ24 plus 2pmol of H-Aβ42) or **l** H-Aβ42/H-Aβ24 mix (8pmol of H-Aβ24 plus 8pmol of H-Aβ42). *N* = 4 to 6 animals per experimental group. Values are normalized on vehicle. Statistical analysis was performed by unpaired T test (*P < 0.05)

To directly investigate whether oligomeric H-Aβ24 forms aggregates retaining Aβ42 and impairing the clearance of Aβ42 through the blood brain barrier (BBB), we used an in vitro BBB transwell model, formed by (brain) endothelial bEnd.3 cells cultivated in the abluminal compartment of cell culture inserts until a post-confluent monolayer had grown (Fig. 7a). Confirmation of the morphological and functional properties of the endothelial cell monolayer (Fig. 7b) were obtained by immunostaining for the tight junction proteins claudin5 (Fig. 7c) and ZO1 (Fig. 7d), connexin43 (CX43) (Fig. 7e) and by the transendothelial electrical resistance (TEER) values reached from day 7 after plating (Fig. 7f). The in vitro BBB model was used to investigate the passage of FAM-labeled or 488-conjugated H-Aβ42 across the endothelial cell monolayer. While the addition of fluorescently labeled H-Aβ42 to the apical side of cell inserts resulted in effective BBB crossing, fluorescently labeled scrambled H-Aβ42 was not efficiently transcytosed (Fig. 7g) thus confirming the reliability and the selectivity of the model as described in literature [50]. Notably, the concomitant presence of H-Aβ24 at the apical side of the cells significantly reduced fluorescently-labeled H-Aβ42 transfer (Fig. 7g, h). No effect of H-Aβ24 on the diffusion of fluorescently labeled scrambled H-Aβ42 was detected (Fig. 7g, h). Similar results were obtained using different Aβ42 concentrations (1 μM or 100 nM Aβ42, Fig. 7g and h). Consistently, H-Aβ42 apical-to-basolateral passage, detected by dot blot analysis of medium recovered from the basolateral compartment, revealed a decreased amount of H-Aβ42 when the latter was pre-incubated with H-Aβ24 at the apical side (Fig. 7i). These data indicate that H-Aβ24 presence results in H-Aβ42 retention, thus reducing its efflux through the BBB and therefore preventing an efficient mechanism of Aβ42 clearance.

Given that H-Aβ24 retains H-Aβ42, thus reducing its clearance through the BBB, lower H-Aβ42 levels in the circulation are expected. For this reason, H-Aβ42 levels were quantified by ELISA assay in serum samples of wt mice 1 week after injection of amyloid species into the brain. As expected, increased Aβ42 peripheral levels were detected in Aβ42 i.c. injected mice (Fig. 7j). Notably, a significant reduction of H-Aβ42 peripheral levels was detected when H-Aβ24 and H-Aβ42 were injected together into the brain at equimolar amounts (4pmol of H-Aβ42 or 2pmol of H-Aβ24 plus 2pmol of H-Aβ42) (Fig 7k). In order to exclude that the lower Aβ42 plasma content, observed after the i.c. injection of H-Aβ24 and H-Aβ42, could result from the reduced amount of Aβ42 injected (2pmol vs 4 pmol), the experiment was repeated upon injection of the same amount of Aβ42, either in association or not with Aβ24 (8pmol of H-Aβ42 or 8pmol of H-Aβ24 plus 8pmol of H-Aβ42). Reduced Aβ42 peripheral levels were detected also in this case when H-Aβ24 was co-injected together with H-Aβ42 (Fig. 7l).

### Injected H-Aβ24 aggregates with endogenously produced mouse Aβ42

**Fig. 8:**
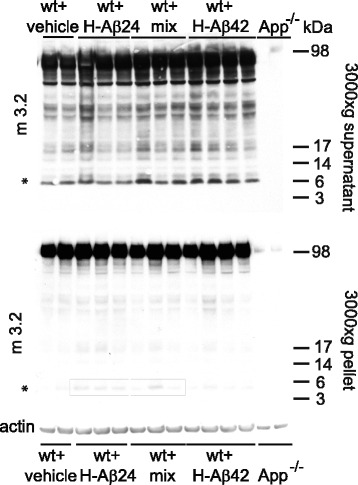
Production of murine-Aβ in H-Aβ24 and H-Aβ24/H-Aβ42 injected mice. Tris-Tricine SDS–polyacrylamide gel electrophoresis (PAGE) followed by immunoblotting using murine-Aβ-specific monoclonal antibody m3.2 of brain homogenate factions (supernatant, *top panel* and pellet, *bottom panel*) of mice injected with vehicle, H-Aβ24, H-Aβ42 or H-Aβ24/H-Aβ42 mix and analyzed after 4 months. Asterisks indicate the presence of 6KDa band positive to m3.2 antibody only in the pellet fraction of mice injected with H-Aβ24. Actin was used as loading control

Considering that H-Aβ24 would reduce Aβ42 clearance thus causing an increase in the levels of brain Aβ42, we hypothesized that deposits observed in mice injected with only H-Aβ24 could derive from a co-aggregation of the injected peptide and endogenously produced mouse Aβ42, retained in the brain. Consistently, immunoblot analysis of brain homogenate fractions using Aβ-specific monoclonal antibody m3.2 (which recognizes residues 10–15 of murine Aβ [51] (Fig. 8; supernatant, top panel and pellet, bottom panel) showed the presence of m3.2 positive bands (squares) in wt mice injected with H-Aβ24 and, at a lesser extent in mice injected with H-Aβ24/H-Aβ42 mix. This result corroborates our hypothesis by which H-Aβ24 injected in the brain interacts with endogenously produced mouse Aβ42. As a specificity control, brain homogenates of mice lacking the amyloid precursor protein APP (App−/−) were negative for the m3.2 antibody (Fig. 8).

### Microglial MMP9 is responsible for Aβ42 degradation and for the production of a C-terminal truncated Aβ fragment

**Fig. 9:**
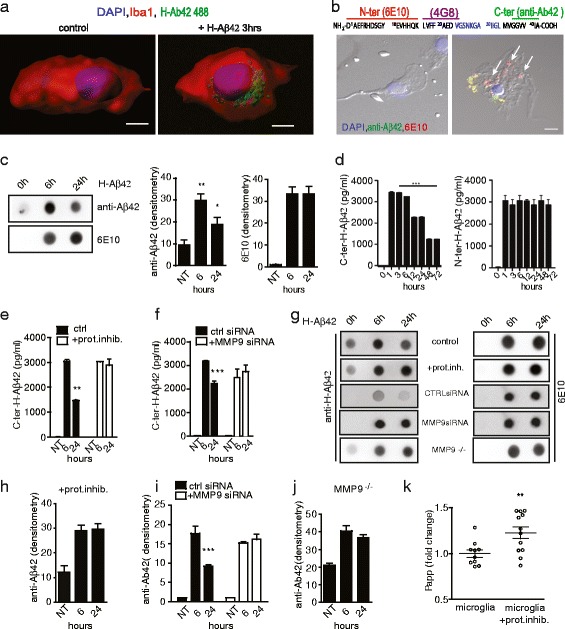
Microglia promote the formation of a C-terminal lacking amyloid fragment in vitro. **a** 3D reconstruction by Imaris software of microglia cells (Iba1 positive, *red*) treated or not for 3 h with 1 μM Aβ42–488 (*green*). Scale bar: 4 μm. **b** Bright field images of microglia cells stained with 6E10 (*red*) or anti-Aβ42 (*green*) antibodies, before (left panel) or after (right panel) 24 h incubation with H-Aβ42. Arrows indicate 6E10 positive puncta not co-localizing with anti-Aβ42 positive domains. Scale bar: 10 μm. On top: schematic representation of H-Aβ42 sequence, showing the binding sites for the different antibodies. **c** Dot blot analysis of the extracellular medium collected 6 and 24 h after microglia exposure to H-Aβ42. Histograms represent the densitometry quantification upon staining with anti-Aβ42 C-terminal antibody (left histogram) or 6E10 N-terminal antibody (right histogram). Intensity values are shown. *N* = 5 independent experiments, statistical analysis was performed by One way Anova, Bonferroni multiple comparison test (**P < 0.01, *P < 0.05). **d** Quantification of Aβ42 extracellular levels in microglia cultures exposed to H-Aβ42 for the indicated time points. Two different ELISA kits based on a capture antibody against the C-terminal (left) or N-terminal (right) domains of the protein were used. *N* = 5 independent experiments. Statistical analysis was performed by One way Anova, Bonferroni’s post hoc test for multiple comparisons (*** *P* < 0.001). **e** ELISA of C-terminal-containing Aβ42 in the extracellular medium of microglia exposed to H-Aβ42 in both control conditions and in the presence of protease inhibitors. *N* = 3 independent experiments. Statistical analysis was performed by two way Anova, Bonferroni’s post hoc test for multiple comparisons. **f** ELISA of C-terminal-containing Aβ42 in the extracellular medium of N9 cells exposed to siRNAcontrol or MMP9siRNA. Statistical analysis was performed by two way Anova, Bonferroni’s post hoc test for multiple comparisons. **g** Representative dot blots of the extracellular medium from H-Aβ42-treated microglia in control conditions, in the presence of protease inhibitors, from siRNAcontrol or MMP9siRNA or from MMP9^−/−^ microglia. Blots are immunostained with anti-Aβ42 or 6E10 antibodies. **h**-**j** Dot blot quantification of the extracellular medium collected from microglia in control conditions or in the presence of protease inhibitors **h**), from siRNAcontrol or MMP9siRNA **i**) or from MMP9^−/−^ microglia **j**) after 6 and 24 h of Aβ42 treatment. **k**) Apical-to-basolateral exchange across the BBB of fluorescent Aβ42 over 120 min upon pre-incubation with Aβ42-treated microglia conditioned medium in the presence or absence of protease inhibitors. Quantification of unidirectional trans-endothelial Aβ42 transport by fluorescence spectrophotometry of *N* = 2 independent experiments; statistical analysis was performed by One way Anova, Bonferroni’s post hoc test for multiple comparisons (**P < 0.01)

In AD patients and in transgenic models of the disease, microglial activation in response to Aβ is followed by Aβ internalization via phagocytosis, in an attempt of restoring homeostatic conditions [52–55]. However, on the other hand, microglia might be detrimental exacerbating Aβ deposition and causing neuronal damage through the production of amyloidogenic, truncated forms of Aβ [12, 54]. To obtain the proof-of-concept that C-terminal truncated fragments could be produced by microglia, we treated primary microglia cultures with 488-conjugated H-Aβ42 and in line with literature, we observed that already after 3 h of treatment Aβ was efficiently internalized by the cells (Fig. 9a). We took advantage of different antibodies, which recognize distinct portions of the protein to assess the formation of H-Aβ42-derived Aβ forms (Fig. 9b). Double immunofluorescence using antibodies against the C-terminal (anti-Aβ42) and the N-terminal (6E10) regions of Aβ42 revealed that, 24 h after its internalization, an antigen becomes detectable inside microglia, which is selectively recognized by the 6E10 (N-ter) but not by the anti-Aβ42 (C-ter) (Fig. 9b arrows).

In line with the formation of a truncated Aβ42 form, dot blot analysis of the extracellular medium of microglia exposed to H-Aβ42 revealed, after 24 h, a reduction of extracellular Aβ42, when stained by the C-terminal anti-Aβ42 (directed against the aa 38–42) but not by the 6E10 antibody, directed against the N-terminal domain of Aβ42 (aa 1–16) (Fig. 9c). We then quantified Aβ42 extracellular concentrations over time using an Aβ42 ELISA kit based on a capture antibody directed against the C-terminal domain of the protein and a detection antibody against aa 11–28. Consistently, we observed a significant decrease of Aβ42 in the microglia medium, starting from 12 h after treatment (Fig 9d, left). On the contrary, an ELISA kit based on the capture antibody 6E10 revealed the lack of changes in the amount of Aβ42 in the medium of microglia (detection antibody 4G8 against the central part of Aβ42, aa 17–24, Fig. 9d, right). Notably, ELISA for the C-terminal domain of the protein revealed that the modifications in the nature of the extracellular amyloid fragments were prevented by microglia incubation with protease inhibitors (Fig. 9e), indicating that microglia promote the metabolic processing of Aβ42, thus favoring the production of fragments which include the N-terminal, but not the C-terminal domain of the Aβ peptide. The lack of changes in the amount of Aβ42 in the medium of microglia, as detected with the antibody 4G8 which recognizes aa 17–24 (Fig. 9d), suggests that the truncated fragment contains the N-terminal part of the protein up to at least aa 23–24, residues which are known to be cleaved by MMP9 [20]. Consistently with the involvement of MMP9 in Aβ42 processing, the production of the C-terminal truncated fragment was inhibited in microglia cells lines (N9) exposed to specific MMP9 siRNA to reduce the enzyme expression (Fig. 9f). The same results were obtained by dot blot staining. Indeed, the exposure of primary microglia to protease inhibitors (Fig. 9g and h), the knocking-down of MMP9 in N9 cells (Fig. 9g and i) as well as the use of primary microglia from MMP9 ^−/−^ mice (Fig. 9g and j) prevented the reduction in the amount of the C-terminal fragment in the medium of cells exposed to Aβ42 for 24 h. Staining with 6E10 antibody revealed the same amount of N-terminal fragments in the extracellular media of cells under the different experimental conditions (Fig 9g, right). To demonstrate that the N-terminal fragments deriving from microglia proteolytic activity are responsible for a diminished Aβ42 clearance in vitro, we performed the same experiment as in Fig. 7g, by incubating the BBB model with microglia conditioned medium (previously treated with unlabeled Aβ42 for 24 h) in the presence or absence of protease inhibitors. As expected, adding microglia conditioned medium significantly reduced 488-conjugated H-Aβ42 clearance across brain endothelial cell monolayers. The presence of protease inhibitors in microglia medium increased fluorescently labelled H-Aβ42 passage through the BBB (Fig. 9k). Overall, these results suggest that microglia play a central role in the production of C-terminal truncated fragments by the activity of extracellular proteases. This phenomenon could be responsible for an enhanced Aβ42 deposition and seeding in the healthy brain.

## Discussion and conclusions

Although the significance of amyloid deposits for the pathogenesis of AD is still under debate [56–58], the observation that, in brain, harmful proteins show high propensity to aggregate indicates that formation of deposits is important in the pathogenesis of brain disorders. While Aβ-containing brain extracts from AD patient or transgenic mouse model have been found to induce Aβ deposition in the healthy brain [8–11, 59], literature evidences clearly indicate that intracranial injection of a single form of synthetic Aβ (Aβ42) does not induce plaque formation in wt brain. Here we show that the injection of a C-terminal truncated synthetic Aβ peptide (Aβ24), which may result from microglial MMP9 proteolytic activity, has seeding properties for aggregate formation in intracranially injected wild type mice. Our results thus provide a direct demonstration of the concept that biologically relevant mixtures of Aβ forms may result in vivo in more complex aggregation dynamics than those predicted by in vitro studies. Notably, amyloid deposits are sporadically detectable also in the non-injected hemisphere and in the hippocampus, thus excluding they may represent post-injectional sprouting or leftover after cortical injection and brain lesioning. Although it is known that the number of amyloid plaques does not necessarily correlate with cognitive impairments ([60–62]; reviewed in [63]), the presence of amyloid deposits at the hippocampal level may explain the occurrence of the behavioral defects observed in injected in healthy mice.

It is now established that protein aggregation takes place, biophysically, once a critical concentration of proteins has been overcome [64]. The lag phase is reduced by the presence of “seeds” [64], which enhance fibrils formation. An efficient process of clearance is required in order to prevent the increase in concentration of “seeds”, which may in turn initiate the aggregation process [64]. Consistently, Aβ clearance rates were found to be impaired in AD patients compared to cognitively normal controls, while there were no differences in Aβ production rates [65]. Although no specific evidence on the clearance of oligomeric forms is currently available, oligomeric Aβ intermediates have been found to alter proteasomal clearance [66].

Several mechanisms for Aβ clearance have been identified, including drainage via the BBB, which is mostly mediated by the low-density lipoprotein receptor related protein-1 (LRP1). LRP1 is localized on the abluminal side of the brain capillary endothelium and mediates Aβ transport across the BBB in the direction of brain to blood [67–69]. When Aβ binds to LRP1 at the brain side of the BBB, a process of transcytosis starts, which mediates rapid Aβ clearance. Notably, LRP1 expression is reduced during aging and in AD as well as in patients with the Dutch-type of cerebrovascular β-amyloidosis. The transcytosis process is very efficient and, indeed, human Aβ injected into different brain regions of wt mice is rapidly recovered in the plasma [70]. Consistently, no plaques nor aggregates are formed in wt brain upon injection of Aβ42 [8, 9]. Interestingly, post-translationally modified forms of Aβ are cleared less efficiently from the brain, like in the case of N-terminal truncated and pyroglutamate-modified Aβ and phosphorylated Aβ [71, 72]. Notably, Aβ peptides with higher β-sheet content are cleared less efficiently from brain, due to a low-affinity LRP/Aβ interaction, mediating brain accumulation of amyloid. Based on the view that insufficient clearance of Aβ plays an essential role in the pathogenesis of AD [73], one may speculate that even low amounts of Aβ forms impairing physiological Aβ clearance and, thereby, increasing Aβ concentration, may foster disease progression [64].

We demonstrate that Aβ24, when concomitantly present with Aβ42 at the abluminal side of the endothelium, slows amyloid clearance through BBB. This is likely due to the fact that H-Aβ24 aggregates, which display higher antiparallel character, retain Aβ42, thus impairing its clearance through the BBB. Although we cannot exclude that Aβ42 levels measured in the serum of injected mice may reflect other routes of clearance besides BBB, our results suggest that the same mechanism may operate also in vivo. In particular, impaired clearance might overcome the critical concentration of proteins initiating the aggregation process and causing the formation of ThT, Congo-red and 6E10-positive aggregates detected in wt brains upon injection of the H-Aβ24/H-Aβ42 mix. Hence, H-Aβ24, and possibly other C-terminal truncated fragments, may function as scaffold proteins to favor both human (in the case of H-Aβ24/H-Aβ42 mix injection) and mouse Aβ42 (in the case of H-Aβ24 injection) recruitment, fibrillation and deposition. Interestingly, Aβ24 has been detected as a major amyloid component in leptomeninges of patients affected by HCHWA-D (hereditary cerebral hemorrhage with amyloidosis, Dutch type). These data indicate that the Aβ24 fragment is in fact formed also in human brain, possibly generated by carboxyl-terminal limited proteolysis [74]. Although future studies will be required to clarify what type of Aβ is deposited, our results are in line with the work of Schlenzig and colleagues which showed that N-terminally truncated and pyroglutamate-modified amyloid beta peptides are less soluble than full-length peptides, increasing aggregation propensity and seeding of amyloid peptides [64, 75]. Notably, wt mice injected with H-Aβ24 alone or with H-Aβ24/H-Aβ42 mix, display, besides Aβ protein deposition, behavioral defects similar to those of age matched APP/PS1 mice.

**Fig. 10:**
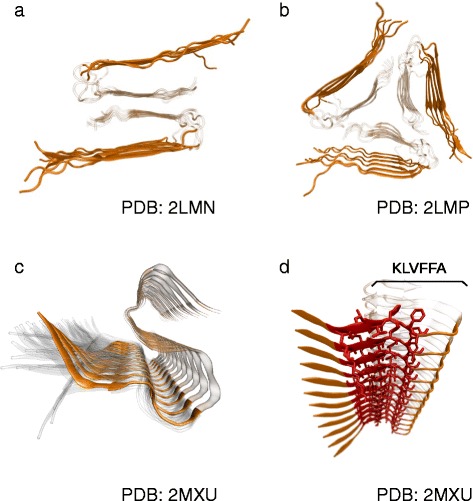
Two-fold **a** and three-fold **b** symmetric structural models reported for Aβ40 by Tycko et al. [94]; and **c** the NMR model by Ishii et al. [89]. Peptide residues 9 to 24 shown in solid *orange*; residues 25 to 40 are transparent; residues 1–8 were not resolved. **d** same as **c**), with the 16–21 KLVFFA region highlighted; KLVFFA has been crystallized by Eisenberg et al. in the antiparallel configuration [79]

One may want to consider whether structural information available for Aβ peptides could provide a possible rationalization of the effect of cleavage at residue 24. The structural arrangement of Aβ fibrils and, even more importantly, oligomers and intermediates is still a matter of debate; structural determinations are made difficult, among other factors, by the presence of extensive polymorphisms [76]. For fibrillar aggregates, numerous studies point towards a β-sandwich motif, i.e. two sheets with strands oriented perpendicular to the long axis of the fibril; β-sandwiches are in turn arranged in higher order structures, i.e. with two-fold or three-fold symmetry around the fibril axis [77]. A common feature of such models is that the two sheets comprise the two separate aggregation-prone regions identified above; therefore, the elimination of the C-terminal region in Aβ24 would delete one of the sheets, and hence be incompatible with the commonly assumed Aβ42 arrangement (Fig. 10).

Although the consensus is weaker for what concerns the structure of oligomeric aggregates and other possible kinetic intermediates, recently proposed models on the basis of NMR data [78] again foresee the presence of cross-β contacts between the two extended amyloidogenic regions identified above. Based on our results and the fact that fibrillar and oligomeric aggregation coexist in a “competitive equilibrium”, one may hypothesize that the presence of Aβ24 shifts the competitive balance towards oligomerization, and away from fibrillation. Structurally, this is consistent with the following data: first, although there is no conclusive structure for Aβ42, NMR-derived models (Fig. 10) have a consensus in attributing the steric zipper (the dry spine of a parallel-β fibril) to the 25–42 C-terminal region of Aβ42, which is of course absent in Aβ24; second, the two algorithms providing a prediction for the parallel/antiparallel character of fibrils (Walsh’s and Tartaglia’s) indicated that Aβ24 still has aggregation potential, a relatively larger antiparallel β propensity and that there is cross-aggregation potential between Aβ1-24 and Aβ25-42. Finally, the 16–21 KLVFFA region is predicted as the fragment’s most amyloidogenic; KLVFFA has been crystallized by Eisenberg and collaborators in the antiparallel configuration [79]. These data are consistent with previous observations that the Aβ24 fragment is present in both monomeric and aggregated forms [55].

Aβ24 represents a prototypical example of a C-terminal truncated fragment generated by Aβ42 proteolysis. Indeed, in line with the concept that Aβ42 is internalized and metabolized by microglial cells [80–82], we provide the evidence that C-terminal truncated fragments can be produced by microglia in a protease-dependent manner. These fragments may share similar properties to those of Aβ24. Among those produced by MMP9 (1–16, 1–20, 1–23, 1–30, 1–33 and 1–34, [19]), one could argue that only Aβ16 should have a different character than the other fragments, because it lacks the 16–20 region which is known to be amyloidogenic and predicted as such by Walsh’s algorithm (among others) for all other proteolytic products. One would therefore argue that, in the 1–16 case, both hypothetical self-aggregation and cross-aggregation pathways would be abolished (Fig. 10). Conversely, C-terminal truncated fragments from the cleavage site at aa residue 20 up to the 34 may possibly share similar properties to synthetic Aβ24. The in vivo relevance of fragments with length possibly exceeding the aa 34 is more questionable, given that fragments produced by microglia lack the C-terminal region, as recognized by the antibody against aa 34–42.

The role of microglia in the production of fragments which could in principle favor plaque formation, like Aβ24, adds an additional detrimental role to microglia in AD. Indeed, an inverse correlation exists between microglia activation and neurodegeneration [83] or cognitive impairment [81, 84], indicating the occurrence of a vicious cycle based on Aβ deposition, inflammation, neuronal damage and cognitive decline. Our evidence demonstrates that microglia may produce, through their metabolic activity, C-terminal truncated Aβ forms which in turn could initiate amyloid aggregation and cause phenotypic defects even in the absence of genetic mutations associated with AD. Here we provide new evidence for the contribution of microglia activation to the development of the sporadic form of AD.

## Materials and methods

### Mice

Mice were housed in the SPF animal facility of Humanitas Clinical and Research Center in individually ventilated cages. Procedures involving animals handling and care conformed to protocols approved by the Humanitas Clinical and Research Center (Rozzano, Milan, Italy) in compliance with national (4D.L. N.116, G.U., suppl. 40, 18-2-1992) and international law and policies (EEC Council Directive 2010/63/EU, OJ L 276/33, 22-09-2010; National Institutes of Health Guide for the Care and Use of Laboratory Animals, US National Research Council, 2011). The study was approved by the Italian Ministry of Health (approval n. 6/2014). All the experimental procedures followed the guidelines established by the Italian Council on Animal Care and were approved by the Italian Government decree No. 27/2010. All efforts were made to minimize the number of subjects used and their suffering. Mice were housed in cages with free access to food and water at 22 °C and with a 12-h alternating light/dark cycle. Double transgenic APPswe/PSEN1dE9 (APP/PS1) mice were purchased from Jackson Laboratory [85]. C57BL/6 J-App (App^−/−^) mice were provided by Hertie Institute and Deutsches Zentrum für Neurodegenerative Erkrankungen (DZNE), Tübingen, Germany. BALB/c Mmp9 < tm1Tvu > (MMP9^−/−^) P1-P3 pups were provided by Istituto Nazionale dei Tumori, Milan, Italy.

### Preparation of synthetic Aβ peptides

Synthetic human Aβ1–42 and Aβ1–24 were purchased from Bachem and prepared as previously described [86] to obtain oligomeric Aβ forms. Briefly, lyophilized Aβ 1–42 and 1–24 were dissolved in dimethyl sulfoxide (DMSO, Sigma) to a concentration of 2 mM and stored in small aliquots at−80 °C. Immediately prior to use, aliquots were quickly resuspended in 50 mM NaPi, 100 mM NaCl pH 7.4 buffer at a concentration of 40 μM, strongly vortexed, sonicated for 30 s and left at room temperature for 20 min before being further diluted for in vivo and in vitro experiments (the concentration used for each experiment is specified in the figure legend). For Aβ clearance experiments, Aβ42–488 (HILyte, AnaspeC) and Aβ42-FAM (Anaspect) were solubilised in NH_4_OH and stored in small aliquots at−80 °C.

### Antibodies

Antibodies used for immunoblot (western/dot blot), immunoprecipitation, and immunofluorescence were as follows: monoclonal antibody 6E10 (1:2000; Covance), which recognizes residues 1–16 of human Aβ; rabbit anti-human beta amyloid 1–42 (1:1000; Alpha Diagnostic International) which recognizes C-terminal 6 aa peptide from human beta 1–42; 4G8 antibody (1:2000; Covance) directed against the central part of Aβ42 (aa 17–24); m3.2 (kindly provided by Prof. Paul Matthews) recognizes residues 10–15 of murine Aβ; rabbit anti-Iba1 antibody (1:500; Wako); ZO-1 (1:1000, clone R40.76, Millipore); Connexin-43 (1:400; C6219, Sigma); Claudin-5 (1:800; ABT45, Millipore). Secondary antibodies (1:200; Alexa Fluor®-conjugated, Molecular Probes).

### Prediction of aggregation propensities

We used the AMYLPRED2 meta-predictor [87] to compare the aggregation profiles of the sequences of full Aβ42 peptide with respect to the truncated form including Aβ residues 1–24 (henceforth Aβ24). The meta-predictor identifies putative amyloigenic regions on the basis of the consensus between 11 methods considering a range of physico-chemical properties. Further analysis was carried out with the algorithms PASTA 2.0 [44] and PAGE/ABSOLUTERATE [88] in order to obtain quantitative predictions of putative aggregation propensities, rates, and the fibrillar’s beta-strand parallel versus antiparallel character. Putative three-dimensional fibrillar arrangements were obtained from the PDB database entries 2LMN, 2LMP, 2MXU [89].

### Thioflavin T (ThT) assays

Thioflavin T (ThT) dye was purchased from Sigma Aldrich. For the ThT assays, 200 μL of Aβ42 (at 8 μM concentration), of Aβ24 (at 8 μM concentration), or of the equimolar mixture of the two peptides (at 4 μM concentration each) were incubated in 50 mM NaPi, 100 mM NaCl pH 7.4 buffer at 37 °C with ThT at 10 μM concentration. At different incubation times, the fluorescence emission spectra of the samples were collected after excitation at 450 nm [45] or at 270 nm [47] by the Cary Eclipse Spectrofluorimeter (Varian Australia Pty Ltd, Mulgrave VIC, Australia). Quartz cuvettes of 1 cm path length were employed. For time course experiments, the samples were kept at 37 °C and analyzed at each time point.

### ATR-FTIR spectroscopy

For the ATR-FTIR measurements, 2 μL of Aβ42 (at 100 μM concentration), of Aβ24 (at 100 μM concentration), or of the equimolar mixture of the two peptides (at 50 μM concentration each) were deposed on the single reflection diamond element of the ATR device (Quest, Specac, UK). Spectra were recorded after solvent evaporation to allow the formation of an hydrated film as previously described [48]. FTIR measurements were performed using the Varian 670-IR spectrometer (Varian Australia Pty Ltd, Mulgrave VIC, Australia) under the following conditions: 1000 scan coadditions, 25 kHz of scan speed, 2 cm^−1^ of spectral resolution, triangular apodization, and a nitrogen-cooled Mercury Cadmium Telluride detector. Fourier self deconvolution was obtained with a full width at half height of 13.33 cm^−1^ and a resolution enhancement factor K = 1.5 [48] using the Resolutions-Pro software (Varian Australia Pty Ltd, Mulgrave VIC, Australia).

### Intracranial injections

Stereotaxic intracranial injections in mice brains were made under a mixture of ketamine (100 mg/kg) and xylazine (10 mg/kg) anesthesia. After surgical exposure of dura mater, both the bregma and the skull surface served as the stereotaxic zero points. Using a Hamilton syringe, 4 μL of H-Aβ or vehicle (vehicle consists of physiological buffer−50 mM NaPi, 100 mM NaCl pH 7.4−) were injected into the neocortex (AP 1 mm, ML 2 mm, DV−2 mm) with a speed of 1.5 μL/min, the needle was kept in place for an additional minute before it was slowly drawn out. For each experiment 4 pmol of H-Aβ42, 4 pmol of H-Aβ24 and 2 pmol of H-Aβ42 plus 2 pmol of H-Aβ24 for the mix condition were injected, as described in [70, 90]. Different conditions were used for specific experiments as specified in the text. After suturing the incision, mice were maintained on a warm pad until recovery from the anesthesia, then returned to their cages. All procedures were conducted in accordance with institutional guidelines for the care and use of experimental animals.

### Behavioral tests

#### Open field

Mice were placed in a multi-unit open field maze (ViewPoint instruments) with field chamber (25 cm long and 25 cm wide), and activity was recorded using ViewPoint video tracking software. Each quadrant was digitally divided into a peripheral and a central region using ViewPoint video tracking software. The central quadrants are collectively referred to as the center zone and peripheral quadrants are collectively referred to as the peripheral zone. Data were collected continually for 30 min and the distance traveled (cm), velocity (cm/s), and the distance traveled in the center zone versus the peripheral zone were all recorded and scored automatically. In the open field task ambulatory movements (valued as distance traveled and movement speeds) and anxiety-like behaviors (as the as distance traveled in the center zone versus the peripheral zone) can be assessed in response to a novel environment.

#### Social interaction task

The social interaction test was used to measure how mice respond to a social partner during a 5-min test following isolation housing. Since isolation housing potentiates expression of innate territorial defensive responses, in this test we evaluated the reaction of mice to the presence of a new animal in free conditions in the same arena. For this test, mice were placed for 30 min alone into the open field arena to familiarize themselves with the new environment. After this time a new female was introduced and the record started. We used the ViewPoint system to count the number of time the tested mouse made contact with the new females. The decision to use females was made to avoid any occurring of an aggressive behaviour simply caused by a normal intermale instinct.

#### Novel object recognition (NOR)

The test apparatus consisted of an open field box measuring 50 cm × 25 cm and all sessions were video-recorded. The first day the animal was allowed to explore the empty field arena for a 10-min time period (habituation session) before being exposed to a 10-min period of familiarization session in the presence of identical objects (A/A). This familiarization session was followed by 1 h and 24 h delays during which the animals were returned to their home cages. After the delay the animals performed 10-min of test session (A/B) in which one object was kept as during the familiarization session (A) and another was changed (B). The objects were made of hard plastic and had previously been counterbalanced to control for any object preference bias. The total amount of time spent with each object was recorded and scored using fully automated ViewPoint video tracking software. The time spent around each object was defined as the time in which the animal directed its nose to the object at a distance <2.0 cm and/or by the animal touching the object with its nose. Data are shown as the total amount of time that animals spend exploring the novel object during both the 1 and 24 h delay.

### Microglia cell culture

Primary microglia were obtained from mixed cultures prepared from the cerebral cortex of mice at the postnatal day 1 (P1-3). Microglia cells were isolated by shaking flasks for 45 min at 230 rpm at day 10 after plating. Cells were then seeded on poly-L-ornithine (Sigma) pre-coated wells at the density of 1,5×10^5^ cell/mL in DMEM containing 20 % heat-inactivated fetal bovine serum (FBS) and incubated at 37 ° C in a humidified atmosphere of 5 % CO2 and 95 % air. Where indicated, cells were pre-treated for 45 min with protease inhibitors and EDTA 2.5 mM (Roche) which were directly added to the cell culture medium. N9 cells were maintained in IDMEM (Gibco Laboratories, USA), supplemented with 10 % FBS. For siRNA transfection, N9 cells were plated at a concentration of at 1×10^5^ cells/mL into either 96 or 24 multiwell plate. Specific siRNAs were diluted at a final concentration of 20 nM siRNA. Cells were used within 48–72 h after transfection. When not differently indicated, for in vitro experiments microglia were treated with H-Aβ42 400 nM.

### Endothelial cells culture and BBB model

Mouse brain endothelial cells (bEnd.3) [BEND3] (ATCC® CRL2299™) were used as a representative BBB model. bEnd.3 cells were cultured at 37 °C, 5 % CO_2_/saturated humidity in DMEM supplemented with 10 % heat-inactivated fetal bovine serum (FBS), 1 % penicillin-streptomycin.

### Transendothelial cell electrical resistance (TEER) assay

bEnd.3 cells were seeded at a concentration of 30000 cells/cm^2^ onto geltrex-coated (thin layer; Gibco) transwell inserts (polycarbonate, 12 mm diameter, 3 μm pore size; Costar) until a monolayer was established. TEER was assessed using a Voltohmeter (Millicell Electrical Resistance System, Millipore). Background resistance from cell-free matrix-coated transwells was subtracted from recorded values to determine absolute TEER values and corrected for the area covered by the cell monolayer. TEER was measured once a day to monitor cell confluence and development of tight junctions. Change in absolute TEER from T_0_ for each individual transwell was recorded over time and then averaged for each day before treatment.

### Aβ42 permeability of the endothelial barrier

To assess Aβ42 exchange across the BBB model, bEnd.3 cells were cultured upside-down on a transwells system as described above, until steady-state TEER had been reached. 1 μM FAM-labeled Human Beta-Amyloid (1–42) or FAM-labeled scrambled Beta-Amyloid (1–42) or HiLyte Fluor™ 488-labeled Beta-Amyloid (1–42) (Anaspec Peptide, Eurogentec) in the presence or absence of Human Beta-Amyloid (1–24) (Bachem) were pre-incubated for 20 min in FluoroBrite™ DMEM (Gibco) medium at 37 °C, and added to the apical (brain) side corresponding to the donor compartment. The basolateral (blood) side of the transwells represented the receiver side and was exposed to medium alone. Alternatively, primary microglial cells were incubated for 24 h at 37 °C in FluoroBrite™ DMEM medium containing Human Beta-Amyloid (1–42) (Bachem) in the presence or absence of protease inhibitors (Roche). Microglia conditioned medium was recovered and spun for 2 min at max speed. 1 μM HiLyte FluorTM 488-labeled Beta-Amyloid (1–42) was pre-incubated for 30 min at 37 °C with microglia conditioned medium, and added to the apical compartment. Following 120 min of incubation at 37 °C with slow mixing, samples were collected from the upper and lower chambers to assess the movement of fluorescently-labelled Aβ42 across the bEnd.3 monolayer (apical to basolateral). The level of fluorescence in the media collected was measured for 488-Aβ42 (λ_ex_ = 503 nm and λ_em_ = 528 nm) or for FAM-Aβ42 (λ_ex_ = 492 nm and λ_em_ = 518 nm) using a Synergy™ H4 Hybrid Multi-Mode Microplate Reader (BioTek). Relative fluorescence units were converted to concentration according to prepared standard, and were corrected for background fluorescence. The amount of fluorescently-labelled Aβ42 was calculated as apparent permeability (P_app_) coefficient [ref. Zhao Z 2015 Nat Neurosci; Keaney J 2015 Sci Adv] and expressed as fold change of control. Briefly, Aβ42 volume cleared (ΔV_c_) was calculated using the equation ΔV_c_ = C_lower_ × V_lower_/C_upper_, where C_upper_ and C_lower_ are fluorescently-labeled Aβ42 concentrations in the donor and receiving compartments, respectively, and V_lower_ is the volume on the basolateral side. The volume cleared (ΔV_c_) was plotted against assay time. Permeability coefficients (P) were calculated by dividing against the surface area of the filter (1.12 cm^2^).

### Immunocytochemistry and cells imaging

Cells were fixed for 15 min at room temperature in a 4 % (w/v) PFA, 4 % (w/v) sucrose, 20 mM NaOH and 5 mM MgCl_2_ in PBS, pH 7.4. Cells were permeabilized and blocked for 30–60 min at room temperature in 15 % (w/v) goat serum, 0.3 % (v/v) Triton X-100, 450 mM NaCl, 20 mM phosphate buffer, pH 7.4 and incubated at 4 °C overnight with primary antibodies diluted in blocking buffer. Coverslips were mounted onto slides with PBS containing 70 % glycerol and 1 μM DAPI. Representative images were taken using a confocal microscope Fluoview FV1000 Olympus IX81 (Center Valley, PA, USA) with an oil immersion objective (×40 or × 60 × 1.4 NA Plan-Apochromat; Olympus) using laser excitation at 405, 488 or 594 nm, and processed using Fiji [91]. Alternatively bEnd.3 cells were stained with Diff Quick (Dade Behering, BioMap) and acquired with inverted microscope Olympus IX53 (Center Valley, PA, USA).

### Brain homogenate preparation and western blot analysis

Animals were anesthetized and perfused with PBS before brains were removed, weighted and homogeneted in a mild hypotonic buffer (50 mM Tris pH 8, 150 mM NaCl, 5 mM EDTA supplemented with phosphatase and protease inhibitor, EDTA-free, Roche). Supernatants were then centrifuged for 1 h at 3000 × g to pellet insoluble material, including insoluble Aβ species. Samples were either frozen on dry ice or LDS sample buffer (Invitrogen) containg 5 % β-mercaptoethanol was immediately added. Pellets were directly resuspended in 20 μl of LDS sample buffer. 10 μl sample were loaded onto a Bolt 4–12 % Bis-Tris Plus Gels (Thermo Fisher) for Western Blot evaluation. Samples were separated using MES buffer and transferred onto a 0.22 μM nitrocellulose membrane (Bio-Rad). Membranes were washed in TBS-Tween (150 mM NaCl, 50 mM Tris and 0.1 % (v/v) Tween-20) and incubated for 45 min at room temperature in blocking solution (5 % milk or 2.5 % serum bovine albumin in TBST). Membranes were subsequently probed overnight at 4 °C with primary antibodies diluted in TBS-T buffer. Membranes were washed extensively and incubated for 45 min at room temperature with horseradish peroxidase-conjugated (HRP) secondary antibody diluted in TBST buffer. Antibody-specific signals were detected using enhanced chemiluminescence reagents (Clarity Western ECL substrate, Bio-Rad).

### Dot blot analysis

Aliquots of supernatant samples (200 μL) were loaded on nitrocellulose membrane Trans-Blot Transfer Medium (0.22 μm, Bio-Rad), by vacuum deposition on the Bio-Dot SF blotting apparatus (Bio-Rad). Serial dilution curves of Aβ42 synthetic protein were preliminarily run to obtain non-saturating condition of immunodetection. DB dots images were analysed by Image Lab™ software (Bio-Rad).

### ELISA

Aβ42 levels were determined using specific ELISA kit (Amyloid-beta (x-42) ELISA IBL, International) following manufacturer’s instructions. Briefly, 100 μL of sample was added into the pre-coated plate and was incubated overnight at 4 °C. After washing each well of the pre-coated plate, 100 μL of labeled antibody solution was added and the mixture was incubated for 1 h at 4 °C in the dark. After washing, chromogen was added and the mixture was incubated for 30 min at room temperature in the dark. After the addition of stop solution, the resulting color was assayed at 450 nm using a microplate absorbance reader (Synergy H4 Synergy™ H4 Microplate Reader, BioTek).

### Histological and immunohistochemical analyses

Mice were anesthetized and perfused with 0.9 % saline, followed by 4 % PFA. The 30 μm cryosections of brain were blocked in PBS containing 10 % goat serum and 0.1 % Triton X-100 for 1 h at room temperature before being incubated overnight at 4 °C with primary antibodies. The following day, the slides were rinsed in PBS and incubated at room temperature for 1 h with secondary antibody. The slides were processed using the ABC detection kit (Vector Laboratories). The presence of the antigens was revealed using the DAB (diaminobenzidine) (brown) peroxidase substrate kit (Vector Laboratories). Immunofluorescence and ThT staining were performed on brain sections. Brain slices were washed three times in PBS and incubated for 1 h at room temperature in blocking solution (3 % BSA). Subsequently, the slides were washed 3 times and incubated overnight with specific antibodies. The next day, sections were washed in PBS and incubated for 2 h at room temperature with specific fluorochrome-conjugated secondary antibodies diluted in 3 % BSA in the dark. ThT solution was prepared as described above. Final solution was added to free floating slides for 1 min and very quickly washed with 80 % methanol followed by 3 washes with distilled water. Images were acquired by Virtual Slides microscope (VS120, Olympus). For quantification, both diffuse-plaques and dense-core plaques were considered, as described in [92]. Small dots and deposits at the slice edge were not counted. The entire surface of the slice was examined. For congo red histological staining, slices were air-dried on glass overnight, then stained with a 0.2 % congo red solution according to [93]. Specimens were acquired by transmittance polarized light microscopy, FITCH filtered epifluorescence. For quantification, bright field images were analyzed by color segmentation plugin-ImageJ software (NIH, Bethesda, MD). The entire area of deposits was considered.

### Statistical analysis

Statistical analysis was performed with PRISM software (Graph-Pad Software, San Diego, CA, USA). Data are expressed as mean ± SEM. Comparisons between two groups were performed using Student’s *t* test or by non-parametric two-tailed Mann–Whitney *U* test. For the comparison of more than two groups, two-way ANOVA followed by Bonferroni’s post hoc test was used. Differences were considered significant at *P < 0.05, **P < 0.01, ***P < 0.001.
